# The gynecological examination in Pantaneiro mares

**DOI:** 10.21451/1984-3143-AR2019-0031

**Published:** 2020-03-17

**Authors:** Bruno Milan, Breno Fernandes Barreto Sampaio, Eliane Vianna da Costa e Silva, Cassia Rejane Brito Leal, Bruna Navarro, Danilo Carloto Gomes

**Affiliations:** 1 Faculdade de Medicina Veterinária e Zootecnia, Universidade Federal de Mato Grosso do Sul, Campo Grande, MS, Brasil

**Keywords:** gynecological examination, pantaneiro horse, uterine biopsy

## Abstract

Gynecological examination is essential to assess the reproductive tract of mares and can provide important information about the uterine environment. It includes physical, vaginal, and rectal examination, ultrasound, cytology, culture, and endometrial biopsy. The present study aimed to perform gynecological examination and fertility to assess the fertility prognosis of Pantaneiro mares that have not been reproductively active and to determine their reproductive ability. Eight mares underwent ultrasound and gynecological examination and artificial insemination. Ultrasound revealed changes only in one mare. Histopathological findings were mild, such as periglandular and perivascular inflammatory cell infiltrates, fibrotic areas, glandular dilation, glandular island formation, and edema due to the phase of the estrous cycle. One animal was classified in category I and the others in category IIA. Cytological changes were found in only one mare. Endometrial culture from five mares resulted in isolation of *Pseudomonas sp., Bacillus sp., Escherichia coli* and *Candida sp.* Only four mares resulted pregnant through artificial insemination, using the same stallion with fresh semen, which has been proving fertility. Thus, mares with better uterine conditions will not always become pregnant and those with mild changes will not always be barren.

## Introduction

**Figure 1:**
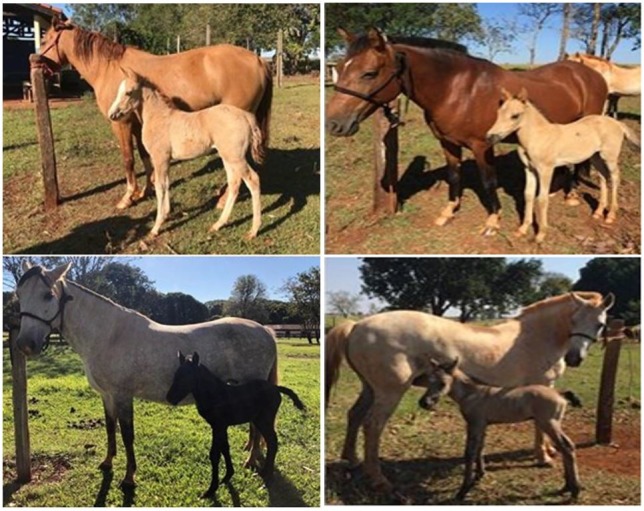
Pantaneiro mares used in the study.

Pantaneiro horse is a breed that is adapted to the extreme environmental conditions of the Pantanal region of Brazil via natural selection (Figure 1). Hooves of this animal can withstand terrains that are flooded for up to 6 months at a stretch. Moreover, the animal can feed from submerged pastures. These characteristics make this animal extremely important as it is used in the management of beef cattle, which is the region’s primary economic activity. This breed had nearly become extinct owing to indiscriminate crossbreeding with other horses. As a result, the Brazilian Association of Pantaneiro Horse Raisers was formed with the aim of devising a conservation and genetic improvement program that was supported by the Federal University of Mato Grosso, the Federal University of Mato Grosso do Sul (UFMS), and the Brazilian Agricultural Research Company (Embrapa), which contribute to the program chiefly by conducting scientific research (Santos et al., 2016).

In equine, the most widely used reproductive biotechnologies include artificial insemination (AI) and embryo transfer (ET) as they are more economic and easier to uses (Chalhoub et al., 1996). Brazil is one of the leading countries that use ET. One of the requisites for this technique to succeed is a thorough examination of the female reproductive system (Carno, 2003).

Gynecological examination is indicated when reproductive mares are being acquired and before starting the breeding, particularly in old mares, in those with a history of reproduction or conception problem, or when no information about the animal’s fertility is available (McKinnon and McCue, 2011).

Ultrasound examination can be performed using a 5- or 7.5-MHz linear transducer. The cervix, uterus body, uterine horns, and ovaries could be transrectally examined. This examination is intended to obtain an image that allows the evaluation of the uterine conditions (body and horns), the assessment of endometrial characteristics, and the determination of cysts, foreign bodies, or liquid inside the lumen. The ovaries should be assessed for their size, the number and size of follicles, the presence or absence of corpora lutea, and any changes in these structures (Ley, 2006).

Endometrial cytology is frequently used to diagnose inflammation (Asbury, 1984; Riddle et al., 2007). It is an inexpensive examination that is also easy to perform and may enable a quick diagnosis of inflammatory processes (Mattos et al., 1984). Mares with normal endometria present low polymorphonuclear leukocyte (PMN) count. When a cytological examination determines three or more PMNs for every five light fields, it´s indicates inflammation (Ley, 2006). Mares with cytological findings of inflammation have a lower probability of becoming pregnant than those with normal cytological findings (Nielsen et al., 2010).

Endometrial biopsy plays an important role in gynecological examination by enabling the evaluation of mare’s reproductive stage, if necessary (Kenney, 1975). Endometrial biopsy allows the evaluation of the structural integrity of the endometrium regarding inflammatory infiltrates, fibrosis, and the dilation of the endometrial glands and lymphatic vessels. It also allows the observation of endometrial variations throughout the mare’s estrous cycle (Love, 2011).

Endometrial culture is used to determine the causative agent of a suspected endometritis and to obtain an antibiogram to guide future treatment. For culturing, sample must be collected prior to cytology or biopsy to avoid contamination (Silva et al., 1987). A diagnosis of endometritis must be made by considering the results of both endometrial cytology and culture owing to the probability of false positive results due to the contamination of the sample by microorganisms from the lower genital tract or by careless manipulation. Samples can be obtained through swabs, uterine lavage, or endometrial biopsy (Troedsson, 2011).

This study aimed to characterize the uterine status and fertility of the Pantaneiro mares of the UFMS herd using ultrasound, cytology, biopsy, and endometrial culture examinations after 3-5 years of sexual inactivity and to evaluate the results regarding the success of introducing young mares and reinstating the other mares to reproductive activity.

## Material and methods

This experiment was approved by the UFMS Ethics Committee on the Use of Animals and was registered under the number 736/2015.

The experiment was conducted at the UFMS Teaching Farm, located in the municipality of Terenos, State of Mato Grosso do Sul, Brazil, latitude 20°26'32”S, longitude 54°51'37”W during January 2, 2018–January 31, 2018.

Gynecological examinations were performed on eight Pantaneiro mares aged 3-29 years, weight: 320-370 kg, height: 1,35-1,40 meters that were owned by UFMS. The examinations were performed during the estrus. Some breeders report that Pantaneiro mares cycle all year long, however our season is held during September through February. Of these, two mares (both 3 years old) had never been in a mating season program. The other mares were reproductively inactive for at least 5 years. So, the eight mares were never used in other experiments.

The mares used in the study were chosen due to their great genetic material and are animals exclusively used for reproduction.

### Ultrasound

Transrectal ultrasound examination was performed using a medisono p3v with 5-MHz transducer. With the animal restrained in a stock, rectal palpation was performed according to the technique described by Carnevale and Olsen (2011).

### Culture

The mares were restrained in a stock to coll ect samples for uterine culture. Their tails were wrapped in cotton bandages, and the perineal region was sanitized with a neutral detergent and povidone-iodine. The samples were collected using the adapted method described by Ley, 2006.

At the laboratory, the swabs were seeded onto plates containing brain and heart infusion agar and blood agar. Then the plates were incubated in an incubatorat 37 °C for up to 72 h and were observed daily. After colony growth was observed, identification was made according to the method reported by Winn et al. (2008) and the Brazilian National Sanitary Surveillance Agency’s *Clinical Microbiology for the Control of Healthcare-Related Infections* manual (Brasil, 2013).

### Cytology

Endometrial cytology was performed in a manner similar to that in the culture with the exception that the swab was replaced with a sterile gynecological brush. The collected sample was performed using the adapted method described by LeBlanc (2011).

### Biopsy

**Figure 2:**
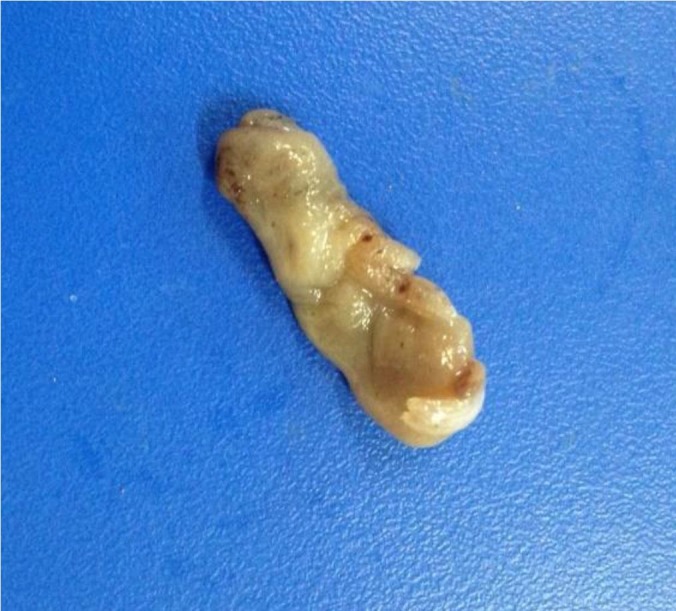
Specimen collected using forceps for specifically conducting uterine biopsy in mares; the fragment is 2-cm long and 1-cm wide.

Uterine biopsy was conducted with the same hygiene as in the culture and cytology examinations. A tissue fragment was extracted using 60-cm biopsy forceps, specific for endometrial biopsy in mares. The collected specimens were 2-cm long and 1-cm wide (Figure 2). The samples were collected using the adapted method described by Love (2011).

### Classification of mares according to biopsy results

The mares were classified according to the scheme proposed by Kenney and Doig (1986), which comprises four categories (I, IIA, IIB, and III). Category I refers to an endometrium that is considered as normal, with little to no changes. Such an endometrium can be considered to have no changes that can reduce the mare’s capability to carry a pregnancy to full term. In category IIA, the endometrium have a few inflammatory cell infiltrates in the compact layer and scattered fibrosis. Mares in this category have a 50%-80% prognosis of full-term pregnancy. Category IIB have an abnormal endometrium with moderate to severe inflammatory cell infiltration in the compact and spongy layers along with significant periglandular fibrosis. These animals have a 10%-50% prognosis of pregnancy. Finally, category III is the category with the highest pathological level, and mares have <10% chance of carrying a pregnancy to term. The endometrium exhibits severe changes, and there is widespread inflammation along with a high degree of periglandular fibrosis. Animals in this category are generally old. Mares that only present widespread inflammation may change to category I or II if the inflammation is treated and resolved. However, animals with a high degree of periglandular fibrosis cannot change categories as fibrosis is an irreversible condition.

### AI

AI was performed in all mares using fresh semen obtained from the same stallion, which has been proving fertility.The mares were monitored through rectal ultrasound to detect the best time for conducting AI, i.e., when the follicle was ≥35 mm in diameter and the uterus had edema 3 or higher the mares were induced with strelin^®^. The insemination was performed one day after induction and ultrasonography was realized one day after insemination to confirm ovulation. The semen was collected using a Botupharma^®^ artificial vagina and was then analyzed for its macroscopic (aspect, color, and volume) as well as microscopic (sperm motility, vitality, and concentration) characteristics. The mares were restrained in a stock and then their perineal regions were sanitized. The semen used in the insemination had 80% (0-100%) motility and vigor 3 (0-4) and the insemination dose was set to 500 × 10^6^ motile sperm, which was administered in the uterine body. Pregnancy was determined 15 days after ovulation.

## Results

Of all mares, only mare 7 presented changes such as accumulation of liquid inside the uterus, which was assessed using ultrasound.

Table 1 shows the results of endometrial culture. Microorganisms were isolated from five animals, with bacterial growth (*Pseudomonas sp., Bacillus sp., Escherichia coli*) observed in the cultures of four of them and fungal growth (*Candida* sp.) in one.

**Table 1:** Results of gynecological examination and artificial insemination in Pantaneiro mares.

**Mare**	**Age**	**Category based on Biopsy**	**Culture**	**Cytology**	**Pregnancy**
1	10 years	IIA	Negative	Negative	Pregnant
2	11 years	IIA	Negative	Negative	Pregnant
3	3 years	I	*Pseudomonas* sp.	Negative	Pregnant
4	10 years	IIA	Negative	Negative	Pregnant
5	10 years	IIA	*Pseudomonas* sp.	Negative	Empty
6	3 years	I	*Bacillus* sp.	Negative	Empty
7	28 years	IIA	*Escherichia coli*	Negative	Empty
8	8 years	IIA	*Candida* sp.	Positive	Empty

**Figure 3:**
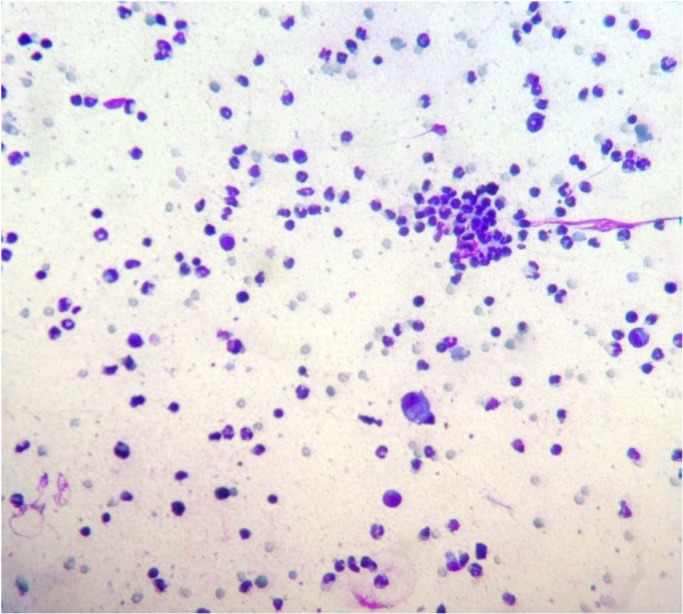
Gynecological examination in Pantaneiro mares. Endometrial cytology (mare 8) shows intense neutrophil infiltration and occasional macrophages. Panoptic staining, 40×.

Only one animal (mare 8) exhibited cytological changes such as a large number of inflammatory cells, comprising neutrophils and some macrophages, and round negative images approximately 1 µm in diameter (Figure 3).

**Figure 4:**
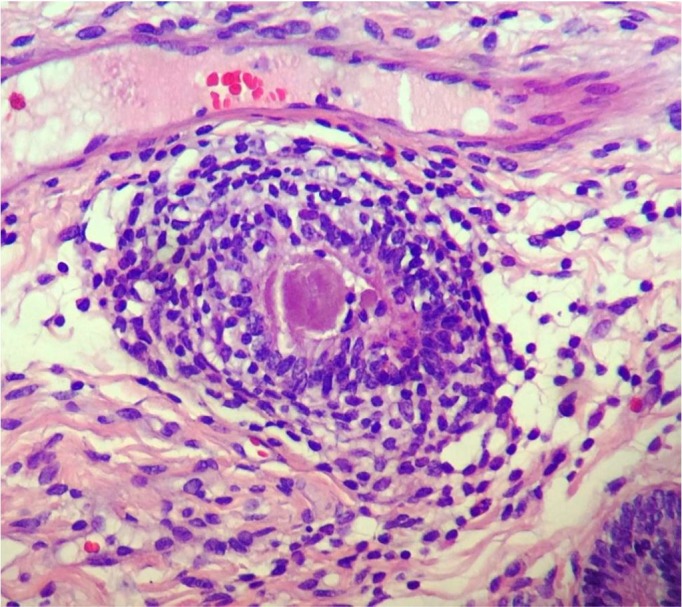
Gynecological examination in Pantaneiro mares. Endometrial biopsy in mare 7 shows lymphocyte infiltration around the gland. Hematoxylin-eosin staining, 40×.

**Figure 5:**
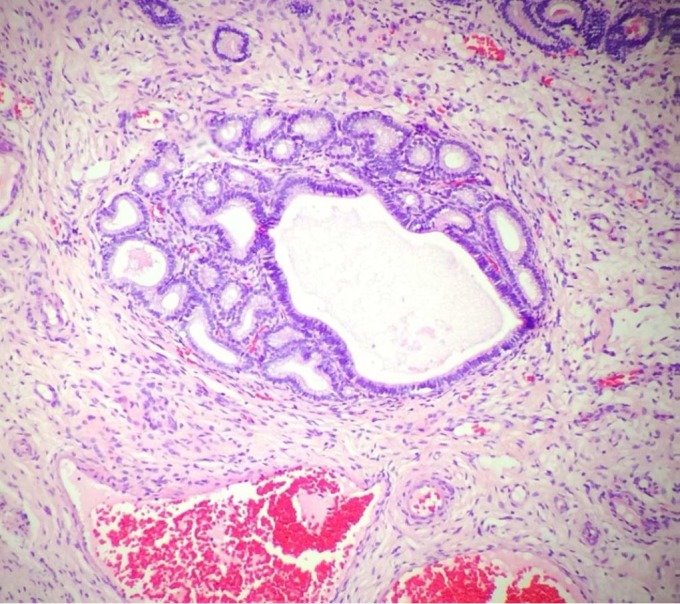
Gynecological examination in Pantaneiro mares. Endometrial biopsy in mare 4 shows moderate proliferation of the fibrous conjunctive tissue, the formation of a glandular island, and glandular dilation. Hematoxylin-eosin, 10×.

**Figure 6:**
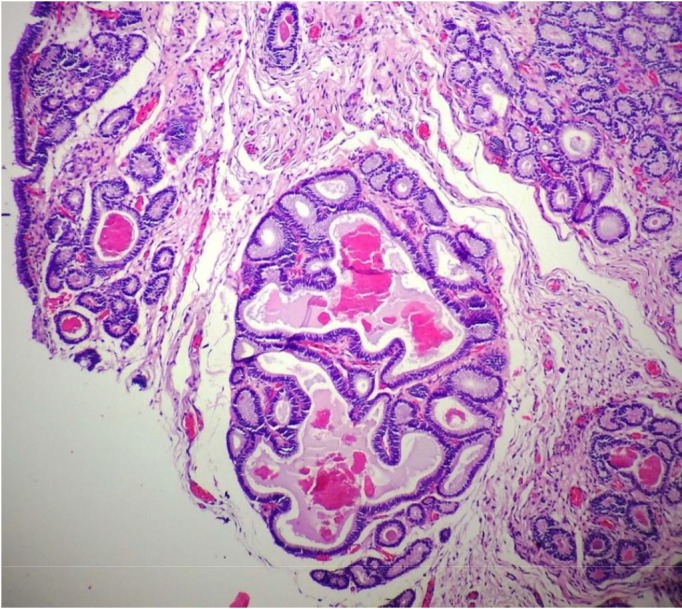
Gynecological examination in Pantaneiro mares. Endometrial biopsy in mare 4 shows moderate proliferation of the fibrous conjunctive tissue, the formation of a glandular island, and glandular dilation. Hematoxylin-eosin, 10×.

**Figure 7:**
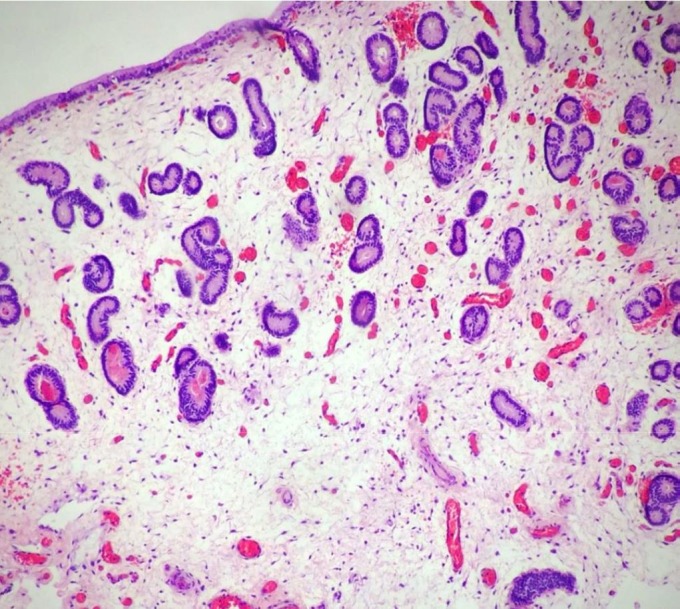
Gynecological examination in Pantaneiro mares. Endometrial biopsy in mare 6 shows intense edema. Hematoxylin-eosin, 10×.

Histopathological examination revealed mild changes, which primarily comprised periglandular (Figure 4) and perivascular inflammatory cell infiltrates and macrophages with a brownish pigment. Few eosinophils were observed in the compact layer, and a small number of lymphocytes and plasmocytes were observed beneath the epithelium. Fibrotic areas, glandular dilation, glandular islands (Figure 5 and 6), lymphatic gaps, and edema due to the phase of the estrous cycle (Figure 7) were also noted. Artifacts were present in all specimens, particularly circulatory hemorrhage artifacts that were observed in all slides. In addition, other artifacts such as congestion, edema unrelated to estrus, and glandular distortion were observed. According to the classification suggested by Kenney and Doig (1986), two mares were classified in category I (mares 3 and 6) and the others in category IIA.

A 50% pregnancy rate was obtained with mares 1, 2, 3, and 4 becoming pregnant.

## Discussion

The results of endometrial culture must always be interpreted in combination with the symptoms and cytological and histopathological findings. This is because if signs of inflammation are detected in biopsy specimens collected from sites close to those where the culture samples were obtained, this will help in increasing the significance of the isolated microorganism (Ley, 2006). When a positive result is obtained in the culture and a negative one in cytology, the possibility of sample contamination during manipulation or even during isolation cannot be ruled out.

Only one animal (mare 8) presented endometrial changes in the cytological examination, with a large number of neutrophils and a small number of macrophages. Round shapes of approximately 1 µm in diameter were observed, which were compatible with fungal structures and were confirmed by the isolation of *Candida* sp., establishing a diagnosis of fungal endometritis. In one study involving 85 mares (Ribas et al., 2014), fungi were detected in five mares, and the authors stated that the culture of the agent combined with cytology are sufficient to establish a diagnosis. *Candida* was also the most frequently isolated genus in the aforementioned study. Animals treated for bacterial endometritis using excessive antibiotics are prone to develop fungal infections (Dascanio et al., 2001).

Uterine biopsy can provide substantial information about the endometrium that cannot be obtained using other techniques. In one of the studied animals (mare 5), siderophages were observed in the spongy layer of the endometrium. Siderophages are macrophages that contain hemosiderin, and their presence indicates an endometrial hemorrhage. This can be observed up to 6 months after delivery, or in the case of this mare, it may be an indication of chronic inflammation (Snider et al., 2011) as the animal had not recently given birth. In two mares (mares 3 and 5), a small number of eosinophils was found in the compact layer. The presence of such cells indicates a specific inflammatory process and is commonly observed in cases of fungal infections or in conditions such as pneumovagina (Snider et al., 2011). Mare 1 presented some PMNs inside vessels, suggesting acute inflammation or indicating the follicular phase of the estrous cycle (Barros and Masuda, 2013). It is up to the professional to evaluate this finding based on other tests.

Two mares (mares 2 and 4) presented periglandular fibrosis. Mare 2 presented a moderate number of lymphatic gaps, which when present in large numbers are considered as a problem. It is considered mild, moderate, and severe when 1-3, 4-6, and >6 layers of fibroblasts are observed (Barros and Masuda, 2013). The formation of glandular islands was observed in mare 4 according to the description by Barros and Masuda (2013), implying a moderate degree of endometrial fibrosis.

Glandular dilation was observed in five animals (mares 1, 2, 4, 5, and 7). The animals were classified according to the number of dilated glands: four animals (mares 1, 2, 5, and 7) had few dilated glands and the condition was classified as mild, whereas one animal (mare 4) had a large number of dilated glands and the condition was classified as moderate. The mechanism underlying glandular dilation is still unclear. It may be a consequence of the constriction of the glandular duct due to fibrosis or a result of the accumulation of a thick secretion, which would increase intraglandular pressure. It has also been suggested that although the endometrium has no muscle cells, a decreased myometrial tone would impair secretion from these glands, leading in their dilation. These hypotheses are not well accepted because only one or two dilated glands are usually found and they do not exhibit a flat epithelium, which would result from pressure. Therefore, the presence of glandular dilation may be associated to a local hormonal disorder (Barros and Masuda, 2013).

According to the classification by Kenney and Doig (1986), none of the mares in the present study met the criteria for categories IIB and III. The only mare that had an endometrium close to normal was mare 6, in which no change of any kind was observed regarding inflammatory infiltrates and fibrosis, and was therefore classified into category I. A study by Eigenheer-Moreira et al. (2007) concluded that the animals that presented the most severe endometritis and fibrosis as histological changes were those with more estrus repetitions. The presence of mild to moderate changes does not necessarily imply that the mare will not be able to carry a pregnancy to term. Moreover, not all mares with more severe changes present reproductive failure (Silva et al., 1987). However, subfertility was directly related to the animal’s age (Eigenheer-Moreira et al., 2007).

Even if the examination results indicate no changes, it is not certain that the animal will conceive, as seen in mare 6, which had the best uterine conditions among all mares in this study but failed to become pregnant. Some factors may be involved such as semen quality, the management of the animals, and feeding. The presence of mild changes does not imply that the mare will fail to conceive, as seen in mares 2, 3, and 4. It can be observed that as the animal ages, the frequency of certain endometrial changes such as fibrosis increases, as seen in mares 2 and 8. Ideally, mares that do not become pregnant should undergo the examinations again, including an antibiogram to seek the best treatment.

In this study was observed that those mares that didn´t present uterine infection and had the uterus classified as IIA were successful in the reproduction program, while the remaining mares classified as IIA, with infection, had a unsuccessful reproduction result.

## Conclusion

It is concluded that even after a prolonged period of sexual inactivity, the uterine status of Pantaneiro mares was not that much impaired. In the gynecological examination there were found some alterations, such as positive cultures, inflammatory cell infiltrates and biopsy changes. Even without any treatment, the pregnancy rate was of 50%. The examinations performed were fundamental to infer the animals’ actual uterine condition.
